# A Neural Network Reveals Motoric Effects of Maternal Preconception Exposure to Nicotine on Rat Pup Behavior: A New Approach for Movement Disorders Diagnosis

**DOI:** 10.3389/fnins.2021.686767

**Published:** 2021-07-20

**Authors:** Reza Torabi, Serena Jenkins, Allonna Harker, Ian Q. Whishaw, Robbin Gibb, Artur Luczak

**Affiliations:** Canadian Centre for Behavioural Neuroscience, University of Lethbridge, Lethbridge, AB, Canada

**Keywords:** data driven analysis, animal behavior, post-natal development, maternal preconception nicotine exposure, deep neural network

## Abstract

Neurodevelopmental disorders can stem from pharmacological, genetic, or environmental causes and early diagnosis is often a key to successful treatment. To improve early detection of neurological motor impairments, we developed a deep neural network for data-driven analyses. The network was applied to study the effect of maternal nicotine exposure prior to conception on 10-day-old rat pup motor behavior in an open field task. Female Long-Evans rats were administered nicotine (15 mg/L) in sweetened drinking water (1% sucralose) for seven consecutive weeks immediately prior to mating. The neural network outperformed human expert designed animal locomotion measures in distinguishing rat pups born to nicotine exposed dams vs. control dams (87 vs. 64% classification accuracy). Notably, the network discovered novel movement alterations in posture, movement initiation and a stereotypy in “warm-up” behavior (repeated movements along specific body dimensions) that were predictive of nicotine exposure. The results suggest novel findings that maternal preconception nicotine exposure delays and alters offspring motor development. Similar behavioral symptoms are associated with drug-related causes of disorders such as autism spectrum disorder and attention-deficit/hyperactivity disorder in human children. Thus, the identification of motor impairments in at-risk offspring here shows how neuronal networks can guide the development of more accurate behavioral tests to earlier diagnose symptoms of neurodevelopmental disorders in infants and children.

## Introduction

Many neurological disorders, such as attention deficit/hyperactivity (ADHD) and autism spectrum disorder (ASD), have an early life onset. Although the successful treatment of the consequence of childhood onset disorders depends upon the early diagnosis of at-risk children (Raza et al., 2020), the methodology related to early diagnosis is underdeveloped. For example, mothers outperform experts in the early diagnosis of conditions such as ASD but the way that they do so is *ad hoc* (Sacrey et al., 2018). Many methods and tools have been introduced in order to address the problem of diagnosis and quantification of human disorders in animal models (Basso et al., 1995; Kabra et al., 2013; Berman et al., 2014; Machado et al., 2015; Wiltschko et al., 2015; Ben-Shaul, 2017; Markowitz et al., 2018; Mathis et al., 2018; Arac et al., 2019; Graving et al., 2019; Pereira et al., 2019). Nevertheless, for animal models and for human childhood disorders, early detection is difficult because symptomology must be detected within the limited motor repertoire displayed by infants (Schamhardt et al., 1993). To address the problem of early diagnosis, we introduce a deep neural network that automatically classifies spontaneous behavior and extracts, in a data-driven way, movements that distinguish control and experimental groups of animals.

We applied our network to study the rat pups born to maternal preconception nicotine exposed (MPNE) mothers. Nicotine is one of the most widely used drug of abuse by preconception parents and it is capable of perturbing many aspects of development (Dwyer et al., 2008; Devoto et al., 2020). Preconception nicotine can influence offspring development *via* three main mechanisms; it may induce physiological changes in the mother that alter the fetal environment, it may induce epigenetic modifications in the oocyte that shape ontology (Bohacek and Mansuy, 2013), and it may change the quality of maternal care, thereby resulting in the behavioral transmission of an altered developmental trajectory. Nicotine also influences brain development, e.g., by interacting with nicotinic acetylcholine receptors (nAChRs), affecting neuronal proliferation, differentiation, and maturation (Dwyer et al., 2008; Blood-Siegfried and Rende, 2010). There is limited research into the effects of MPNE on behavior (Holloway et al., 2007; Vassoler et al., 2014; Zhu et al., 2014; Yohn et al., 2015; Renaud and Fountain, 2016) and currently no studies consider its impact on early postnatal development. Therefore, the current research addresses two gaps in our understanding of nicotine’s impact on early infant behavior. First, does nicotine administration during the preconception period in prospective dams, as opposed to the prenatal, preconception + prenatal, or paternal preconception period, affect behavior? Second, are offspring affected at an early stage of infant development, thus demonstrating an early impact of MPNE on offspring locomotion and its sensitivity to experimental detection?

To address these questions, we first analyzed neonatal (10-days-old) rat pup video recordings using standard locomotor-derived kinematic measures. Then we showed that a neural network can improve on this conventional analysis by identifying causative symptomology of the effect of MPNE. Importantly, we also present how to extract knowledge from the deep neural network in order to identify novel behavioral components that distinguished the nicotine exposed group from the control group.

## Results

### Effect of Maternal Nicotine Exposure Prior to Conception on Offspring: Analyses of Behavior Using Expert Selected Measures

**FIGURE 1:**
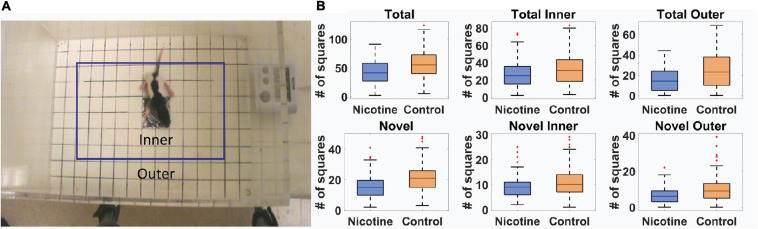
Movement of 10-day old rat pups in open field task. **(A)** Definition of outer and inner portions of the open field. **(B)** Movement measures in nicotine and control animals reveals significant effect of MPNE on offspring exploration (see Methods for measures description). On each box, the central mark indicates the median, and the bottom and top edges of the box indicate the 25th and 75th percentiles, respectively. The whiskers extend to ±3SD, and red marks show data points outside of 3SD range.

Standard “exploratory” locomotor measures were used to investigate the effect of MPNE on offspring locomotor development (Methods). Of 351 rat pups, 191 were from preconception sucralose-exposed dams, and 160 were from preconception nicotine-exposed dams (Methods). Ten day old (P10) pups were placed singly in the open field for one minute and their behavior was videotaped to investigate locomotor development (Methods). The movement of an animal was described in terms of two movement kinematics (Mychasiuk et al., 2013; Jenkins et al., 2018). (1) Total activity was the total number of square entries for either front paw of the animal during exploration. (2) Novel activity was the number of unique square entries, which relates to locomotor complexity. Those measures were calculated separately for the inner and outer part of the open field (Figure 1A, Methods). A statistical comparison of the above movement measures of the MPNE (nicotine exposed dam) and control groups (sucralose-exposed dam) are shown in Figure 1B. The MPNE group was less active, entered fewer squares and explored fewer novel squares than did the control group (Total_*Control*_ = 57.0 ± 1.8, Total_*Nicotine*_ = 42.2 ± 1.6; Total Inner_*Control*_ = 32.0 ± 1.2, Total Inner_*Nicotine*_ = 26.4 ± 2.0; Total Outer_*Control*_ = 24.0 ± 1.2, Total Outer_*Nicotine*_ = 15.0 ± 1.0; Novel_*Control*_ = 21.0 ± 0.6, Novel_*Nicotine*_ = 15.5 ± 0.6; Novel Inner_*Control*_ = 11.1 ± 0.4, Novel Inner_*Nicotine*_ = 9.1 ± 0.3; Novel Outer_*Control*_ = 9.9 ± 0.5, Novel Outer_*Nicotine*_ = 6.3 ± 0.3; “±” represents SEM; *p* < 0.001 for all comparisons using *t*-test; using non-parametric Mann-Whitney U test also gave significant results for all comparisons with *p* < 0.003). We did not detect significant sex differences on any of the above measures (*t*-test, *p* > 0.05 for all measures). In summary, on all measures the MPNE offspring showed less exploration than the control offspring.

### Combining Movement Measures to Distinguish the Control vs. Nicotine Groups

As described above, we quantified the behavior by using typical kinematic measures employed in an open field task. This approach requires assumptions regarding which features of the behavior will be useful in distinguishing between treatment groups. To estimate the reliability of these expert selected features, we used machine learning algorithms to predict treatment groups using all six values of behavioral measures described above. We used five different algorithms to ensure that our results were not dependent on a specific data analysis method. For all algorithms we used fivefold cross-validation, where we trained the model on 80% of trials and predicted the treatment group for the remaining 20% of trials. We repeated this process 5 times to predict group category for every trial. The algorithms discriminated between the two groups with accuracy between 57–64% (Decision tree: 57%; Random forest: 61%; Logistic regression: 61%; K-nearest neighbors: 63%; Support vector machine: 64%) (Supplementary Text 1). This means that based on described movement measures it is possible to tell with about 64% accuracy if it is a control or nicotine group animal (chance level is 50%). We then applied principle component analysis (PCA) to the movement measures. The distribution of points for both classes largely overlapped in PC space (Supplementary Figure 1). It indicates a weak discriminability between classes, which is consistent with the above result using machine learning algorithms.

### Using Deep Neural Network to Distinguish the Control vs. Nicotine Groups

**FIGURE 2:**
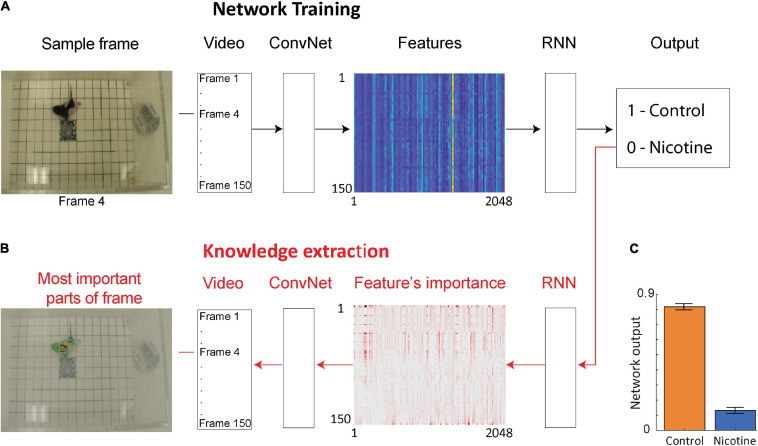
Neural network architecture for data-driven analyses. **(A)** The network is trained using video clips of single trials (each consisting of 150 video frames). Frames are then passed through a convolutional network (ConvNet) to extract 2,048 high level image features from each frame. The features from 150 successive video frames are then given as an input to a recurrent neural network (RNN) to analyze temporal information across frames. Based on this information, RNN predicts a group category for each video clip (Output). **(B)** After the network is trained, information is extracted from the network weights in order to identify image features and the parts of each video frame that were the most important to network decision making. For visualization, only every 20th feature is shown. **(C)** Average activity of the output neuron for animal videos from each group.

To investigate if additional information could be extracted from the rat pup’s behavior, we used a deep neural network to examine the same videos of MPNE and control animals in the open field task (Figure 2A). This approach does not require specifying which behavioral measures should be used. Rather, the neural network discovers by itself which features in video (e.g., shapes, movements, etc.) are the most predictive of the treatment groups. Specifically, we used a convolutional network (ConvNet) (Szegedy et al., 2016) to convert each video frame (400 × 350 pixels) to a set of 2,048 features. Those features may loosely correspond to object edges. Features from 150 video frames from a single video clip were then combined and passed to a recurrent neural network (RNN). This allowed the analysis of animal movements throughout each trial (1 trial = 1 video clip consisting of 150 frames corresponding to 50 s). The network was then trained to assign a correct group category to each video clip (Figure 2A), and then information was extracted from the network to investigate its decisions (Figure 2B, see next section). After training, the network was able to distinguish videos of the MPNE and control groups with 87% accuracy. This accuracy is higher than the classification accuracy obtained from kinematic defined movement features (57–64%). Figure 2C shows the average activity of the output neuron for the control group (mean = 0.82 ± 0.02 SEM) and the nicotine group (mean = 0.13 ± 0.017 SEM). The activity of the output neuron was bounded between 0–1, with 1 corresponding to the control category. For example, a value of the output neuron of 0.9 can be interpreted as the network indicating that it is 90% “confident” of identifying a control animal, and only 10% “confident” that it is a MPNE animal. For calculating the network’s prediction accuracy, values of the output neuron above 0.5 was considered as identifying a rat pup in the control group, and values below 0.5 as the rat pups in the MPNE group.

To verify that our network does not require fine parameter tuning for robust performance, we also tested four variations of the network. In particular, we modified the number of neurons and layers in the RNN, and we repeated the training and testing on the same data. The modified networks produced results similar to those of the original network (Supplementary Figure 2). To ensure that network accuracy is not a result of an overfitting and that our network can generalize to new animals, all predictions were obtained using fivefold cross-validation as described above. Thus, no videos of the predicted animal were included in the training dataset. Altogether, these results indicate that there is information about MPNE in the behavior that is not accounted for by the standard movement analyses.

### Extracting Knowledge From the Neural Network

Considering that the network classified the animal groups from videos with higher accuracy than the kinematic measures, we investigated what movement features were the most informative for the network. We applied a recently developed Layer-wise Relevance Propagation method (LRP) to extract knowledge from deep neural networks (Bach et al., 2015; Lapuschkin et al., 2019) (Methods). First, we identified which features extracted from the videos were contributing the most to the predictions made by the RNN (Figure 2B–features importance array), and then we investigated which parts of each frame corresponded to those most informative features (Figure 2B—left side). This knowledge extraction method reveals the network’s focus for decision making.

**FIGURE 3:**
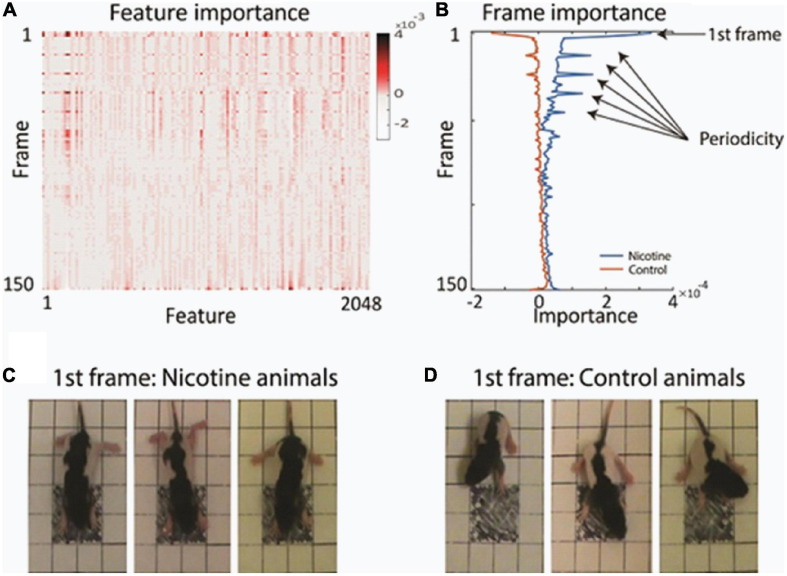
Finding the most informative behavioral features used by the network for decision making. **(A)** Average feature importance over all videos. **(B)** Average importance of each video frame, for each animal group. This revealed that the 1st frame and every 11th frame were particularly important. **(C,D)** Typical starting postures (1st frame) of MPNE **(C)** and control animals **(D)**. Examples of 3 rats from each group are shown. For visualization clarity, only the portion of frame with the pup is shown. Note extended legs in the MPNE group, and collected legs supporting weight in the control group.

Examination of the feature’s importance to the matrix revealed that certain video frames were particularly informative for the network decision. For example, the first frames had multiple features which contributed to network classification more strongly than subsequent frames (Figure 3A and Supplementary Figure 3). Plotting the average value of the features separately for each animal group showed the high discriminative power of those initial frames (Figure 3B). To investigate why the first video frames were singled out, we closely inspected those first frames. We found that on average, there was a meaningful difference in the starting posture and starting movement between the MPNE and control animals. Figures 3C,D illustrates the difference in their starting posture as soon as the pups in the open field box. The MPNE animals sprawled, with the fore and hind legs extended, whereas the control animals had their limbs beneath in a posture of supporting the body. In short, the MPNE animals displayed reduced postural support. The lack of postural support indicated by extended limbs could also be observed as the MPNE animals initiated movement. Once moving, the temporal features of movement were also different between MNPE and control animals. Notably, the control animals began to move as soon as they were placed in the open field. They collected their body by bringing their limbs to a weight bearing posture and made small lateral movements of their head as they initiated movement. The MPNE animals mostly lingered (not moving), then took more time to establish postural support and only then initiated movement.

**FIGURE 4:**
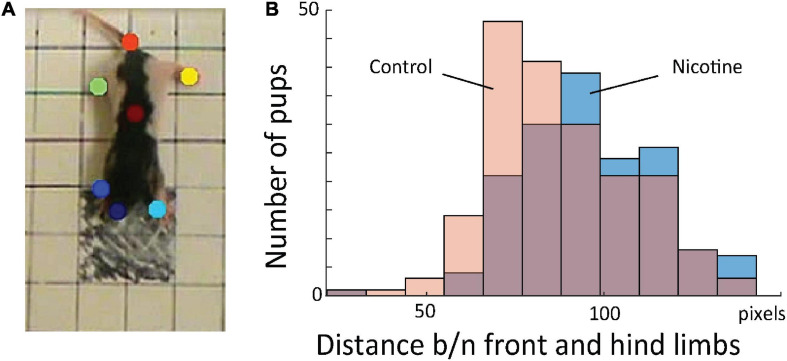
**(A)** Sample frame with semi-automatically superimposed virtual markers on body parts. **(B)** Distribution of distance between fore and hind limbs in the first frames for all animals. This shows that MPNE animals (blue bars) had their legs more extended, indicating reduced postural support as comparing to control pups (pink), a result consistent with that shown in Figures 3C,D.

Because knowledge extraction from the network revealed that the initial posture is a highly discriminative feature between MPNE and control pups, we developed measure to quantify it. For that, first, we used DeepLabCut software (Mathis et al., 2018), which allowed for semi-automatic marking of the position of multiple body parts (four legs, nose, tail base and center of the body; Figure 4A). Next, from x and y coordinates of resulted marks, we estimated pose by calculating the average distances between front and hind limbs in the initial frame. Consistent with our visual observation, MPNE animals had displayed a significantly larger distance between front and hind limbs as compared with controls, indicating reduced postural support (DistNicotine = 96.2 pixels ± 1.53 SEM, DistControl = 87.57 pixels ± 1.46 SEM, p < 0.0001, t-test; Figure 4B).

**FIGURE 5:**
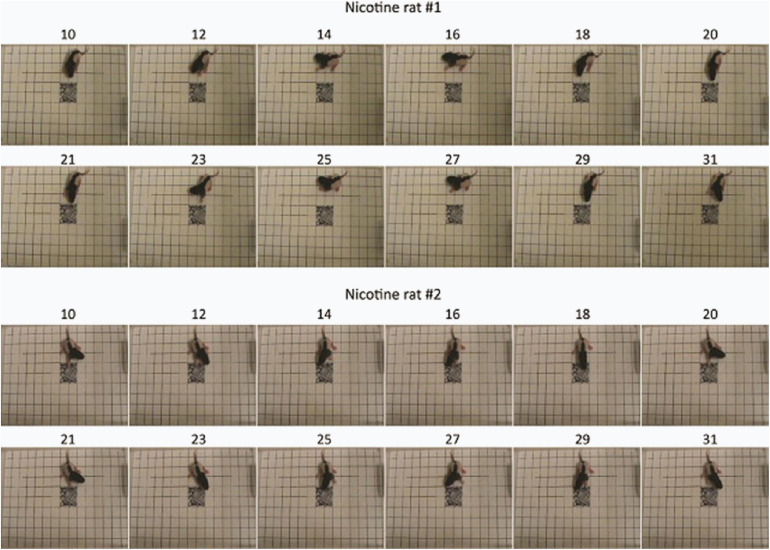
Stereotypical behavior in two rat pups from the MPNE group. Numbers above the pictures represent the frame number. Note the almost exact same body position every 11 frames.

**FIGURE 6:**
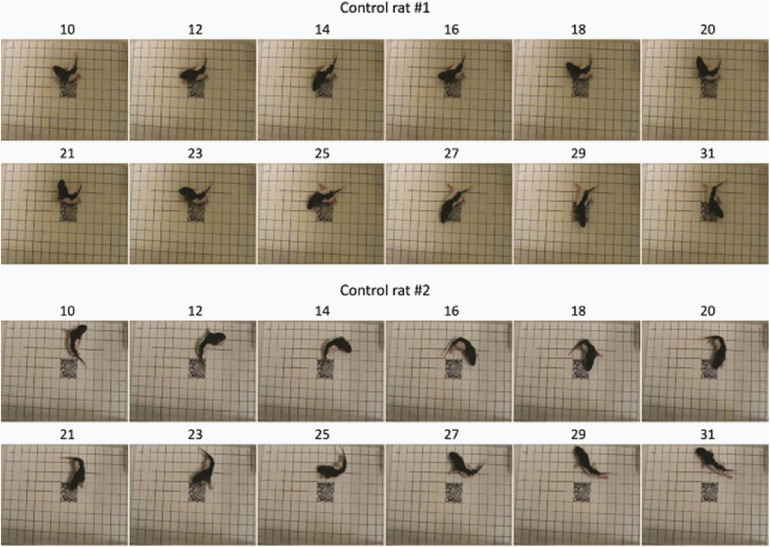
Sample behavior of typical control animals. The movements are less repetitive compared to nicotine animals and more diverse as exemplified by pivoting (rat #2).

Visualization of feature importance also showed unexpected periodic movement changes (Figure 3B). Specifically, features occurring about every 11th frame, corresponding to period of about 3.7 s, were informative for the network’s distinction between the MPNE and the control groups. This was also confirmed with spectral analyses shown in Supplementary Figures 4, 5. To investigate the behavior underlying the distinguishing movements, we divided the videos into 11 frame segments and aligned the segments (Figure 5). This revealed a stereotypical, repetitive behavior in MPNE animals. The animals made repeated lateral movements that returned the animal to its initial position. For comparison, Figure 6 illustrates a typical temporal sequence of two control animals at the same time. The control animals also make lateral movements, but the amplitude and frequency of movement are different from that of the MPNE animals. For example, in the control rat #1, the lateral head movement begins at frame 10 and it ends at frame 18. Its next lateral movement increases in amplitude, thus modifying the sequence of movement (i.e., frames 21 and 31 are not the same in Figure 6 for rat #1). Moreover, some of the control animals pivoted as part of the lateral movement (Figure 6, rat #2). Note that although our analyses showed that features of importance peak at frames 10, 21, …, etc., it should not be interpreted that only those specific frames are of significance to the network. Rather, it should be seen as an indication that at those times the network recognized a periodic stereotypical sequence. Thus, the network identified from raw video data a stereotypical behavior as a distinguishing feature of MPNE pups.

**FIGURE 7:**
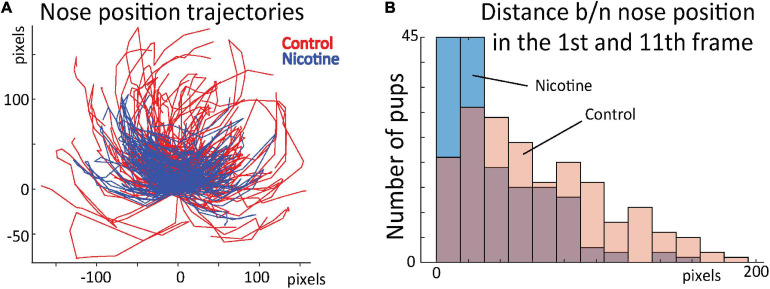
**(A)** Position of rat nose during first 16 frames for control (red) and MPNE pups (blue). Each animal is represented by a line connecting 16 points. For visualization, only every 2nd animal is shown. All trajectories are aligned such that nose position in frame 1 is set to point (0, 0). **(B)** Distribution of distances between nose position in frame 1 and 11. The shift of distribution to the left for the MPNE group shows that nicotine exposed pups were more likely to return to the same position after first sequence of movements.

To test whether the network-uncovered differences in stereotypical behavior distinguished the control and nicotine groups, we conducted additional analyses. Using DeepLabCut marks (Figure 4A), we tracked nose position for the first 16 frames for all rats (Figure 7A). Next, we calculated distance between nose position in the 1st and 11th frame. This allowed us to estimate the relationship between the starting position of the 1st and 2nd sequence of movements. Consistently with results presented in Figures 5,6, the average distance for MPNE group was significantly smaller (mean DistContr = 37.7 pixels ± 2.9 SEM, DistNicotine = 60.4 pixels ± 3.1 SEM; p < 0.0001, Kolmogorov-Smirnov test; Figure 7B). Repeating the analyses using the 3rd and 14th frame gave similar results. This confirmed greater stereotypy in making repeated movements in nicotine exposed group.

We also tested whether only the coordinates of body parts marked with DeepLabCut could provide better features than the ConvNet for predicting the animal group. For that, in all video frames we tracked the position of the nose, limbs, tail base and body center as illustrated in Figure 4A. All points corresponding to frames from one trial were combined as one input to the RNN (similar to ConvNet features in Figure 2; RNN with 256 LSTM units). Thus, each video frame was represented by x- and y-coordinates of seven marked body parts. This procedure resulted in a 62% accuracy in distinguishing the control and MPNE animals. This is a lower accuracy than using original videos (87%), suggesting that ConvNet features selected in a data-driven way contain additional information useful for behavioral classification. Increasing the number of selected body parts for digitization may result in improved RNN performance relative DeepLabCut features. It is noteworthy, however, that the advantage of our network is that it can directly predict movement deficits from raw videos and does not require human decisions on which body parts to select.

## Discussion

The neural network described here revealed motoric impairments in at-risk rat pups whose mother received preconception nicotine. Impairments include a reduction in postural support, slower maturation of warm-up movements, and stereotype in the component movements of warm-up. These motoric abnormalities in the development of the rat pups may be the first symptoms of what might later become abnormalities in adult behavior. Thus, they may be diagnostic of early symptoms of conditions analogous to those of human developmental disorders. The network analysis also provided insights into the hypoactivity of the MPNE pups, as their reduced posture, slow warm-up and stereotype would be expected to compete with locomotion. Thus, by analyzing the network’s decision-making process, new insights into behavioral differences were obtained. We suggest that this network methodology could be useful for the analysis of behavior of other animal analogs of human movement disorders as well as for the analysis of at-risk human infants.

### Significance of Behavioral Results

Our data-driven approach identified impaired postural support, reduced warm-up and increased stereotypical behavior of warm-up components of the MPNE pups. The interpretation of the results of our network analysis capitalized on previous work showing that the development of infant rat movement is organized. When an adult rat is placed on a horizontal surface in an open environment it sequentially makes lateral, forward and dorsoventral movements that escalate in amplitude into forward locomotion (Golani et al., 1981; Golani, 1992). Such warm-up behavior is also a feature of the ontogeny of motor development, in which the topographic dimensions of movement emerge and escalate as maturation proceeds (Golani et al., 1981). Thus, in terms of warm-up, the MPNE animals display lower level of maturation, featuring reduced postural support and movement relative to the control pups.

The infant MPNE pups also displayed stereotyped behavior in that once making one lateral head movement they then repeated the movement at regular intervals rather that escalating warm-up movements into locomotion. Stereotype, as featured in tics, repetitive movements and compulsive behavior, is a feature of many developmental conditions including ASD and ADHD. Stereotype is also symptomatic of use of drugs of abuse and many adult neurological conditions, especially those that affect the basal ganglia (Eilam et al., 2006; Lelard et al., 2006; Singer, 2009; Wolgin, 2012; Berman, 2018; Martino and Hedderly, 2019). That the infant MPNE rat pups displayed stereotype suggests that the MPNE treatments resulted in neural changes in the rat pups that may be analogous to those that produce stereotype in human conditions. Future work could examine both the neural basis of MPNE-based stereotype as well as its influence on adult motor and cognitive behavior.

In the present study we also used conventional locomotor measures with the MPNE rat pups and found that the pups displayed reduced locomotion. A reduction in locomotion can have many causes but our network results suggest that reduced postural support, immaturity in warm-up and stereotype could all contribute. Thus, an important feature of the network analysis is that it pointed to potential first causes of the more behaviorally-holistic symptomology of locomotor measures. Many tools have been constructed for the diagnosis of developmental disabilities, but most are compromised by questions related to reliability. The reliability of measurements of activity changes in conditions such ADHD is illustrative (Egger and Emde, 2011; Wolraich et al., 2019). The utility of more detailed methodology for symptom detection is that it improves the reliability of some behavioral measures and may actually serve as a more valid replacement.

Nicotine exposure during development has long been associated with changes in locomotor behavior. Prenatal, preconception + prenatal, and paternal preconception nicotine have all been linked with hyperactivity in young adult offspring that can propagate through multiple generations (Bruin et al., 2010; McCarthy et al., 2020). However, here we report a decrease in locomotion following maternal preconception nicotine exposure. An important distinction between the study presented here and previously reported results is the age at which the pups were tested; we analyzed the emergence of locomotor behavior in 10-day-old pups. To our knowledge, no other studies have explored the impact of preconception nicotine on young offspring locomotion. One study examined the effects of prenatal nicotine on 19-day-old offspring and found a similar decrease in activity in the nicotine-exposed pups compared to control pups (LeSage et al., 2006). Furthermore, they report that pups prenatally exposed to nicotine were less active than controls during the initial exploration of the open field but were equally active after 10 min of exploration. This finding is reminiscent of our observation that MPNE pups had slowed warm-up and increased stereotypy relative to control pups. Therefore, it may be that the effects of nicotine on locomotor development are age-dependent; nicotine may delay maturation in early life leading to decreased locomotion, but eventually lead to hyperactivity in later life. Further research is required to understand the effects of the cross-generational effects of maternal and biparental preconception nicotine exposure on the emergence of locomotion in early life.

### Comparison With Other Methods

One approach to improving the analysis of exploratory behavior is the use of tracking systems such as those using markers that are automatically or manually attached to body parts (Zhou et al., 2008; Parmiani et al., 2019). In the last few years several methods such as LocoMouse (Machado et al., 2015), DeepLabCut (Mathis et al., 2018), JABAA (Kabra et al., 2013), Optimouse (Ben-Shaul, 2017), and LEAP (Pereira et al., 2019) and DLCAnalyzer (Sturman et al., 2020) have been developed to allow users to identify key points in videos, such as the location of a paw, and then automatically track the movement of those key points across video frames. For instance, in DeepLabCut (Mathis et al., 2018), the experimenter manually labels body parts (e.g., the snout, left ear, right ear, and tail) in selected video frames using virtual markers, and then the network is trained to automatically predict the position of those body parts in the rest of the video. Although this method is useful, it requires investigator decisions about relevant body parts, and it requires separate analyses to determine whether the measures are relevant. Here we have shown that using whole frame video is informative about behavior that a selective investigator may not have predicted a priori. Nevertheless, DeepLabCut did play a valuable role in quantifying the behavior allowing us to validate the results we obtained from the knowledge extraction method. Thus, our network approach adds to the armamentarium of behavioral analysis.

The second category of automated methods such as MoSeq (Wiltschko et al., 2015), MotionMapper (Berman et al., 2014) and B-SOiD (Hsu and Yttri, 2020) first reduce the dimensionality of the video data, and then relate the results to the behavioral components. These methods require image pre-processing and proper image alignment, and additional methods must be applied for classifications. Our method also offers an alternative to these approaches. First, the convolutional network works with raw images without the need of pre-processing and without the difficult task of image alignment, and second, the network automatically identifies the most relevant behavior for predictions. In short, our approach offers a one-step solution for feature selection and animal group classification.

The method presented here also provides significant advancement on our previous network used for analyses of the skilled reaching behavior of stroke rats (Ryait et al., 2019). Specifically, here we introduced analyses in a time dimension, which allowed us to identify repetitive movements. As movement timing is crucial component of animal behavior, the temporal analyses presented here can help to provide more sensitive measures of neurological deficits.

We suggest that the analysis used here could be applied to the behavior of at-risk human infants in the way that it was applied here to infant rats. The development of behavior of human infants is also organized, and this organization is widely used to assess the attainment of developmental milestones (Harris and Heriza, 1987; Sacrey et al., 2020). The assessment of milestones, however, depends upon the accuracy of the rating tool, the expertise of the rater, and it can be confounded by the normal developmental variability of infants. Nevertheless, brief video records of infant behavior could be subject to network analysis to confirm development milestones and to pinpoint the significance of variability as was done here for the behavior of infant rats.

In the future, the behavioral analyses described here could be combined with histological analyses (Faraji et al., 2013) or with electrophysiological recordings (Schjetnan and Luczak, 2011; Ponjavic-Conte et al., 2012; Schjetnan et al., 2019). Most neuronal analyses rely on using expert selected features of brain activity (e.g., spike timing, correlations, firing rate in specific time) to relate it to behavior or sensory stimuli (Luczak et al., 2004; Luczak and Narayanan, 2005; Quiroga and Panzeri, 2009). Applying the present here data-driven approach to electrophysiological data may uncover novel features of neuronal activity patterns, more predictive of animal behavior.

In conclusion, the experimental results answer the questions proposed in the introduction of this study. Nicotine administration during the preconception period in prospective dams altered the behavior of the infant offspring by reducing locomotion, reducing postural support, slowing the development of warm-up, and inducing stereotype in component movements of warm-up. The MPNE offspring were affected at an early stage of infant development, thus demonstrating an early impact of MPNE on offspring locomotion and its sensitivity to experimental detection. These findings suggest that the measurement of infant behavior using a neural network analysis can improve the identification of behavioral irregularities in at-risk infant rats and in the same way, it could be applied to the early identification of signs of symptomology in at-risk human infants.

## Materials and Methods

### Animals

Procedures were conducted in accordance with the Canadian Council of Animal Care and were approved by the University of Lethbridge Animal Care and Use committee. Animals were given food and water ad libitum and were maintained on a 12-h light/dark schedule (lights on from 07:30 to 19:30) in a temperature- and humidity-controlled (21°/50%) breeding room. A total of 45 female Long Evans born in-house from 11 different litters were used. Nicotine-exposed dams (n = 23) received 15 mg nicotine hydrogen tartrate salt (Sigma) per liter of drinking water sweetened with 1% sucralose to increase palatability (Nesil et al., 2011; Collins et al., 2012). Control dams (n = 22) received 1% sucralose only. Nicotine was administered for seven consecutive weeks beginning in adulthood (90-days-old); 7 weeks is the length of the spermatogenic cycle in male Long Evans rats and was chosen to mirror the complementary paternal studies. Nicotine consumption was calculated as mg of nicotine per kg of body weight. The volume of water consumed each day was measured by weighing the water bottles at the same time each day and dividing the change in volume by the number of females with access to the bottle. The mg of nicotine consumed was then calculated from the volume divided by the average weight of the females with access to the bottle. On average, nicotine-exposed dams consumed 2.4 mg of nicotine per kg of body weight per day across the 7 weeks. Females were bred with non-drug-exposed male Long Evans rats (n = 45) the day following completion of nicotine administration. Animals in this analysis were pups from 32 successful litters for a total of 351 pups. Eighteen litters (191 pups: 102 female and 89 male) of the animals were from sucralose-exposed dams, and 14 litters (160 pups: 76 female and 84 male) were from nicotine-exposed dams. Females in both conditions reared their own litters (i.e., pups that were not cross-fostered) until pups were weaned on postnatal day 22.

### Behavioral Testing

Pups were tested in the open field task on post-natal day 10. The testing apparatus was a clear Plexiglas box measuring 20 cm × 30 cm with a grid of 150 squares (10 squares × 15 squares) on the floor each with a size of 2 cm × 2 cm (Figure 1A). Pups were placed individually in the center four squares (shaded black) and left to explore the box for 1 min while being recorded from above. The open field was cleaned with Virkon between animals.

Kinematic movement measures and their definitions in the scoring procedure are as follow:

Novel = the number of unique squares that either front paw of the pup enters, up to a maximum of 146 (i.e., the box is divided into 150 squares total (10 × 15), minus the four shaded squares).

Total = the total number of square entries for either front paw (i.e., the number of times a front paw goes from one square to another).

Novel Inner = the number of unique squares in the inner portion of the field that either front paw of the pup enters. (i.e., the number that are within the 6 × 11 squares in the center of the box, minus the four shaded squares).

Novel Outer = the number of unique squares in the outer portion that either front paw of the pup enters. (i.e., the two rows of squares that make up the perimeter of the open field).

Total Inner = the total number of square entries in the inner portion for either front paw.

Total Outer = the total number of square entries in the outer portion for either front paw.

### Deep Neural Network Training and Architecture

For training the ConvNet neural network we used 351 videos: 160 from MPNE animals, and 191 videos from control animals. The original frame rate was 30 frames per second with resolutions 720 × 480 pixels. However, to reduce the amount of data, we used only every 10th video frame (three per second). From each video we excluded the initial period showing the experimenter’s hand releasing the pup. The 50 s of recording (150 frames) after that was used.

The general network architecture is shown in Figure 2. First, a pre-trained convolutional neural network (ConvNet) known as Inception-V3 (Szegedy et al., 2016) was used to extract 2,048 features from each video frame. This reduced each video to a 2D matrix of the size (2,048 features × 150 frames). This matrix was then given as an input to the recurrent neural network (RNN) to predict animal groups. We used a RNN composed of 256 long short-term memory (LSTM) units, which allowed for the extraction of temporal relations between frames. The LSTM layer was followed by a dropout layer of 0.2 to prevent overfitting, and then a dense layer with two neurons with the softmax activation function classified the animal’s behavior. We used “Group K-Fold” in Keras to split the data randomly and uniformly (to prevent the train and test data being biased) into 5 classes. Each run is initiated with random set of weights. Batch size was 100 and Adam optimizer was used with binary cross entropy as the loss function. The code for our network including all parameters is available in the Github repository as Behaviour_Recognizer toolbox: https://github.com/rezatorabi13/Behaviour_Recognizer.

### Knowledge Extraction Method

After the network was trained, information was extracted from the network weights in order to identify image features and the parts of each video frame that most contributed to the network decision. For this knowledge extraction from the network, we used the Layer-wise Relevance Propagation (LPR) method (Bach et al., 2015; Lapuschkin et al., 2019) available in the DeepExplain package (Braitenberg and Schüz, 1998). This method uses the strength of synaptic weights and neuronal activity in the previous layer to recursively calculate the contribution (importance) of each neuron to the output score. Because our network is composed of two parts, ConvNet and RNN (Figure 2A), we first investigated which features were most informative for the RNN to classify animal groups (Figure 2B middle panel). Next, we propagated feature importance back to pixels in the video through the Inception V3 network (Figure 2B left panel, Supplementary Figure 6, and Supplementary Text 2). This provided us with information related to which parts of the image the network was “attending to” when making classifications. This allowed for a check on whether the network was using rat movements rather than spurious features, such as the amount of light, to discriminate between the treatment groups. Using other methods for knowledge extraction like gradient-based methods (Shrikumar et al., 2017; Ancona et al., 2018) gave qualitatively similar results.

## Data Availability Statement

The datasets presented in this study can be found at the following link: https://github.com/rezatorabi13/Behaviour_Recognizer.

## Ethics Statement

The procedures of the animal study were approved by the University of Lethbridge Animal Care and Use Committee in accordance with the Canadian Council of Animal Care.

## Author Contributions

RT, SJ, IW, RG, and AL: conceptualization. SJ and AH: data acquisition and data scoring. RT, AL, and IW: analyses and interpretation of data. RT, SJ, AH, IW, RG, and AL: writing, review, and editing. All authors contributed to the article and approved the submitted version.

## Conflict of Interest

The authors declare that the research was conducted in the absence of any commercial or financial relationships that could be construed as a potential conflict of interest.

## Supplementary Material

Supplementary Figure 1 (A) Principal component (PC) space of behavioral measures. Each dot represents a single animal with MPNE group denoted in blue and control group in orange. Note that more control animals have high values of PC1 in comparison with the nicotine animals. To investigate this, we calculated the correlations between principal components and movement measures (B). We found that the first principal component has the largest correlation with the “Novel” followed by the “Total” measure. This indicates that control animals have a greater tendency to explore new places (i.e., enter a greater number of unique squares) as well as to explore more (i.e., enter more squares overall) than MPNE animals.

Supplementary Figure 2 Different architectures of recurrent neural network (RNN) tested. For this manuscript we selected the top network (one layer of LSTM with 256 neurons). However, all tested networks produced similar results, as shown in the table. The network performance was also robust to changes in video preprocessing. Specifically, down-sampling video by taking every 9th frame instead of every 10th frame (Methods) gave similar accuracy of ∼89%. This shows that our network does not need fine tuning to outperform machine learning methods using expert selected movement measures. (B) Sample learning curve from training top RNN with one layer of 256 long short-term memory (LSTM) (Hochreiter and Schmidhuber, 1997; Greff et al., 2017) units. Note that on training data we achieved 100% accuracy (red line), however, on testing data accuracy was reduced (green line). This suggests that with larger dataset performance of the network may further improve.

Supplementary Figure 3 Most informative features for network decisions. (A) Average of feature’s importance over all videos, as shown in main text in Figure 3A. Considering reoccurring peaks in feature importance, it is apparent that the network is identifying periodic behavior, especially in the early 50 frames (∼17 s), to distinguish MPNE animals from control animals. Our RNN network was able to detect a repetitive pattern in the behavior because it is composed of the LSTM units which have memory and are specialized in identifying sequences of activity. (B) Average relevance (importance) of each 2,048 features across all video data. Average relevance was obtained by averaging columns in the matrix shown in panel (A). (C) The same average feature importance as in (B), but sorted from highest to the lowest value. It illustrates that about 20 features had a disproportional effect on network decision making.

Supplementary Figure 4 Power spectra analysis. In order to investigate periodic behavior shown in Figure 3B, we calculated power spectra of the average feature importance. Blue and orange lines denote MPNE and control animals, respectively. Peak in power spectra at frequency of 0.27 Hz confirmed that video features oscillate with a period of 1/0.27 = 3.7 s. Note that this periodic behavior was seen mostly in MPNE animals. This indicates that MPNE animals have much more stereotypical behavior, while control animals have more diverse and less repetitive movements.

Supplementary Figure 5 Power spectral analysis for 20 most important features. The blue and orange lines indicate MPNE and control animals, respectively. After identifying the most important features as illustrated in Supplementary Figure 4C, the power spectral for each of the 20 top features was calculated in each video. Then, we averaged spectra of each feature, separately for MPNE and control animals, which lead to the 20 graphs shown above. As can be seen, the periodic behavior with the frequency of about 0.27 Hz is clearly visible in nearly all of the features.

Supplementary Figure 6 (Left column) Representative video frames, and the same frames with superimposed network focus (Right column). Red color scale denotes the most informative pixels used by the network to make decisions. This allowed us to verify that the network used features related to rat posture to discriminate control from MPNE animals. This analyses also ensures that the network does not “cheat” by using spurious features like the clock display. To superimpose pixel importance on the frames, the values of importance were rescaled to range 50–250. The pixel importance was obtained by using LRP method described above.

Supplementary Text 1 Applying machine learning algorithms on movement measures.

Supplementary Text 2 Notes on knowledge extraction from neural networks.

## Abbreviations

ADHD: attention-deficit/hyperactivity disorder
ASD: autism spectrum disorder
MPNE: maternal preconception nicotine exposure.

## References

[B1] Alber M. Lapuschkin S. Seegerer P. Hagele M. Schutt K. T. Montavon G. (2019). iNNvestigate Neural Networks! J. Mach. Learn. Res. 20 1–8.

[B2] Ancona M. Ceolini E. Oztireli C. Gross M. (2018). Towards Better Understanding of Gradient-based Attribution Methods for Deep Neural Networks. New York: Cornell University.

[B3] Arac A. Zhao P. Dobkin B. H. Carmichael S. T. Golshani P. (2019). DeepBehavior: a Deep Learning Toolbox for Automated Analysis of Animal and Human Behavior Imaging Data. Front. Syst. Neurosci. 13:20. 10.3389/fnsys.2019.00020. 31133826 PMC6513883

[B4] Bach S. Binder A. Montavon G. Klauschen F. Muller K. R. Samek W. (2015). On Pixel-Wise Explanations for Non-Linear Classifier Decisions by Layer-Wise Relevance Propagation. PLoS One 10:e0130140. 10.1371/journal.pone.0130140 26161953 PMC4498753

[B5] Barnickel T. Weston J. Collobert R. Mewes H. W. Stumpflen V. (2009). Large scale application of neural network based semantic role labeling for automated relation extraction from biomedical texts. PLoS One 4:e6393. 10.1371/journal.pone.0006393 19636432 PMC2712690

[B6] Basso D. M. Beattie M. S. Bresnahan J. C. (1995). A sensitive and reliable locomotor rating scale for open field testing in rats. J. Neurotrauma. 12 1–21. 10.1089/neu.1995.12.1 7783230

[B7] Ben-Shaul Y. (2017). OptiMouse: a comprehensive open source program for reliable detection and analysis of mouse body and nose positions. BMC Biol. 15:41. 10.1186/s12915-017-0377-3 28506280 PMC5433172

[B8] Berman G. J. (2018). Measuring behavior across scales. BMC Biol. 16:23. 10.1186/s12915-018-0494-7 29475451 PMC5824583

[B9] Berman G. J. Choi D. M. Bialek W. Shaevitz J. W. (2014). Mapping the stereotyped behaviour of freely moving fruit flies. J. R. Soc. Interface 11:20140672. 10.1098/rsif.2014.0672 25142523 PMC4233753

[B10] Blood-Siegfried J. Rende E. K. (2010). The long-term effects of prenatal nicotine exposure on neurologic development. J. Midwifery Womens Health 55 143–152. 10.1016/j.jmwh.2009.05.006 20189133 PMC2998347

[B11] Bohacek J. Mansuy I. M. (2013). Epigenetic inheritance of disease and disease risk. Neuropsychopharmacology 38 220–236. 10.1038/npp.2012.110 22781843 PMC3521963

[B12] Braitenberg V. Schüz A. (1998). “Cortical architectonics”. In Cortex: statistics and Geometry of Neuronal Connectivity. (Germany: Springer). 135–137. 10.1007/978-3-662-03733-1_27

[B13] Bruin J. E. Gerstein H. C. Holloway A. C. (2010). Long-term consequences of fetal and neonatal nicotine exposure: a critical review. Toxicol. Sci. 116 364–374. 10.1093/toxsci/kfq103 20363831 PMC2905398

[B14] Ciresan D. Meier U. Schmidhuber J. (2012). “Multi-column Deep Neural Networks for Image Classification” in 2012 Ieee Conference on Computer Vision and Pattern Recognition (Cvpr). (United States: IEEE Computer Society). 3642–3649.

[B15] Collins A. C. Pogun S. Nesil T. Kanit L. (2012). Oral nicotine self-administration in rodents. J. Addict. Res. Ther. S2:004. 23264883 10.4172/2155-6105.S2-004 PMC3527900

[B16] Collobert R. Weston J. Bottou L. Karlen M. Kavukcuoglu K. Kuksa P. (2011). Natural Language Processing (Almost) from Scratch. J. Mach. Learn. Res. 12 2493–2537.

[B17] Devoto F. Zapparoli L. Spinelli G. Scotti G. Paulesu E. (2020). How the harm of drugs and their availability affect brain reactions to drug cues: a meta-analysis of 64 neuroimaging activation studies. Transl. Psychiatr. 10 1–11. 10.1007/bf03274122 PMC7736294 33318467

[B18] Dwyer J. B. Broide R. S. Leslie F. M. (2008). Nicotine and brain development. Birth Defects Res. C. Embryo. Today 84 30–44. 18383130 10.1002/bdrc.20118

[B19] Egger H. L. Emde R. N. (2011). Developmentally sensitive diagnostic criteria for mental health disorders in early childhood: the diagnostic and statistical manual of mental disorders—IV, the research diagnostic criteria—preschool age, and the Diagnostic Classification of Mental Health and Developmental Disorders of Infancy and Early Childhood—Revised. Am. Psychol. 66:95. 10.1037/a0021026 21142337 PMC3064438

[B20] Eilam D. Zor R. Szechtman H. Hermesh H. (2006). Rituals, stereotypy and compulsive behavior in animals and humans. Neurosci. Biobehav. Rev. 30 456–471. 10.1016/j.neubiorev.2005.08.003 16253329

[B21] Faraji J. Gomez-Palacio-Schjetnan A. Luczak A. Metz G. A. (2013). Beyond the silence: bilateral somatosensory stimulation enhances skilled movement quality and neural density in intact behaving rats. Behav. Brain Res. 253 78–89. 10.1016/j.bbr.2013.07.022 23871611

[B22] Golani I. (1992). A Mobility Gradient in the Organization of Vertebrate Movement - the Perception of Movement through Symbolic Language. Behav. Brain Sci. 15 249–266. 10.1017/s0140525x00068539

[B23] Golani I. Bronchti G. Moualem D. Teitelbaum P. (1981). Warm-up Along Dimensions of Movement in the Ontogeny of Exploration in Rats and Other Infant Mammals. P. Natl. Acad. Sci. Biol. 78 7226–7229. 10.1073/pnas.78.11.7226 6947283 PMC349230

[B24] Graving J. M. Chae D. Naik H. Li L. Koger B. Costelloe B. R. (2019). DeepPoseKit, a software toolkit for fast and robust animal pose estimation using deep learning. ELife 8:e47994. 31570119 10.7554/eLife.47994 PMC6897514

[B25] Greff K. Srivastava R. K. Koutnik J. Steunebrink B. R. Schmidhuber J. (2017). LSTM: a Search Space Odyssey. IEEE Transac. Neur. Netw. Lear.Syst. 28 2222–2232. 10.1109/tnnls.2016.2582924 27411231

[B26] Harris S. R. Heriza C. B. (1987). Measuring infant movement: clinical and technological assessment techniques. Phys. Ther. 67 1877–1880. 10.1093/ptj/67.12.1877 2446339

[B27] Hochreiter S. Schmidhuber J. (1997). Long short-term memory. Neural. Comput. 9 1735–1780. 9377276 10.1162/neco.1997.9.8.1735

[B28] Holloway A. C. Cuu D. Q. Morrison K. M. Gerstein H. C. Tarnopolsky M. A. (2007). Transgenerational effects of fetal and neonatal exposure to nicotine. Endocrine 31 254–259. 10.1007/s12020-007-0043-6 17906372

[B29] Hsu A. I. Yttri E. A. (2020). B-SOiD: an Open Source Unsupervised Algorithm for Discovery of Spontaneous Behaviors. bioRxiv 770271. 10.1101/770271 PMC8408193 34465784

[B30] Jenkins S. Harker A. Gibb R. (2018). Maternal Preconception Stress Alters Prefrontal Cortex Development in Long-Evans Rat Pups without Changing Maternal Care. Neuroscience 394 98–108. 10.1016/j.neuroscience.2018.10.023 30366025

[B31] Ji S. Yang M. Yu K. (2013). 3D convolutional neural networks for human action recognition. IEEE Trans Pattern Anal. Mach. Intell. 35 221–231. 22392705 10.1109/TPAMI.2012.59

[B32] Kabra M. Robie A. A. Rivera-Alba M. Branson S. Branson K. (2013). JAABA: interactive machine learning for automatic annotation of animal behavior. Nat. Methods 10 64–U87. 23202433 10.1038/nmeth.2281

[B33] Krizhevsky A. Sutskever I. Hinton G. E. (2017). ImageNet Classification with Deep Convolutional Neural Networks. Commun. Acm 60 84–90. 10.1145/3065386

[B34] Lapuschkin S. Binder A. Montavon G. Muller K. R. Samek W. (2016). The LRP Toolbox for Artificial Neural Networks. J. Mach. Learn. Res. 17:1.

[B35] Lapuschkin S. Waldchen S. Binder A. Montavon G. Samek W. Muller K. R. (2019). Unmasking Clever Hans predictors and assessing what machines really learn. Nat. Commun. 10:1096. 30858366 10.1038/s41467-019-08987-4 PMC6411769

[B36] Le Q. V. Zou W. Y. Yeung S. Y. Ng A. Y. (2011). “Learning hierarchical invariant spatio-temporal features for action recognition with independent subspace analysis” in 2011 Ieee Conference on Computer Vision and Pattern Recognition (Cvpr). (United States: IEEE).

[B37] Lelard T. Jamon M. Gasc J.-P. Vidal P.-P. (2006). Postural development in rats. Exp. Neurol. 202 112–124. 10.1016/j.expneurol.2006.05.018 16814770

[B38] LeSage M. G. Gustaf E. Dufek M. B. Pentel P. R. (2006). Effects of maternal intravenous nicotine administration on locomotor behavior in pre-weanling rats. Pharmacol. Biochem. Behav. 85 575–583. 10.1016/j.pbb.2006.10.012 17141848 PMC1820587

[B39] Luczak A. Hackett T. A. Kajikawa Y. Laubach M. (2004). Multivariate receptive field mapping in marmoset auditory cortex. J. Neurosci. Methods 136 77–85. 10.1016/j.jneumeth.2003.12.019 15126048

[B40] Luczak A. Narayanan N. S. (2005). Spectral representation—analyzing single-unit activity in extracellularly recorded neuronal data without spike sorting. J. Neurosci. Methods 144 53–61. 10.1016/j.jneumeth.2004.10.009 15848239

[B41] Machado A. S. Darmohray D. M. Fayad J. Marques H. G. Carey M. R. (2015). A quantitative framework for whole-body coordination reveals specific deficits in freely walking ataxic mice. Elife 4:e07892. 26433022 10.7554/eLife.07892 PMC4630674

[B42] Markowitz J. E. Gillis W. F. Beron C. C. Neufeld S. Q. Robertson K. Bhagat N. D. (2018). The Striatum Organizes 3D Behavior via Moment-to-Moment Action Selection. Cell 174 44–58. 10.1016/j.cell.2018.04.019 29779950 PMC6026065

[B43] Martino D. Hedderly T. (2019). Tics and stereotypies: a comparative clinical review. Parkinsonism Relat. Disord. 59 117–124. 10.1016/j.parkreldis.2019.02.005 30773283

[B44] Mathis A. Mamidanna P. Cury K. M. Abe T. Murthy V. N. Mathis M. W. (2018). DeepLabCut: markerless pose estimation of user-defined body parts with deep learning. Nat. Neurosci. 21 1281–1289. 10.1038/s41593-018-0209-y 30127430

[B45] McCarthy D. M. Lowe S. E. Morgan T. J. Cannon E. N. Biederman J. Spencer T. J. (2020). Transgenerational transmission of behavioral phenotypes produced by exposure of male mice to saccharin and nicotine. Sci. Rep. 10 1–14. 32686722 10.1038/s41598-020-68883-6 PMC7371742

[B46] Montavon G. Orr G. B. Müller K.-R. (2012). “Neural Networks: tricks of the Trade : second Edition” in Lecture Notes in Computer Science. eds Montavon G. Orr G. B. Müller K.-R.. (Berlin: Springer). 10.1007/978-3-642-35289-8_1

[B47] Mychasiuk R. Harker A. Ilnytskyy S. Gibb R. (2013). Paternal stress prior to conception alters DNA methylation and behaviour of developing rat offspring. Neuroscience 241 100–105. 10.1016/j.neuroscience.2013.03.025 23531434

[B48] Nesil T. Kanit L. Collins A. C. Pogun S. (2011). Individual differences in oral nicotine intake in rats. Neuropharmacology 61 189–201. 10.1016/j.neuropharm.2011.03.027 21504750 PMC3105211

[B49] Parmiani P. Lucchetti C. Bonifazzi C. Franchi G. (2019). A kinematic study of skilled reaching movement in rat. J. Neurosci. Methods 328:108404. 10.1016/j.jneumeth.2019.108404 31445116

[B50] Pereira T. D. Aldarondo D. E. Willmore L. Kislin M. Wang S. S. H. Murthy M. (2019). Fast animal pose estimation using deep neural networks. Nat. Methods 16 117–125. 10.1038/s41592-018-0234-5 30573820 PMC6899221

[B51] Ponjavic-Conte K. D. Dowdall J. R. Hambrook D. A. Luczak A. Tata M. S. (2012). Neural correlates of auditory distraction revealed in theta-band EEG. Neuroreport 23 240–245. 10.1097/wnr.0b013e3283505ac6 22314684

[B52] Quiroga R. Q. Panzeri S. (2009). Extracting information from neuronal populations: information theory and decoding approaches. Nat. Rev. Neurosci. 10 173–185. 10.1038/nrn2578 19229240

[B53] Raza S. Sacrey L.-A. R. Zwaigenbaum L. Bryson S. Brian J. Smith I. M. (2020). Relationship between early social-emotional behavior and autism spectrum disorder: a high-risk sibling study. J. Autism Dev. Disord. 50 2527–2539. 10.1007/s10803-019-03977-3 30852785

[B54] Renaud S. M. Fountain S. B. (2016). Transgenerational effects of adolescent nicotine exposure in rats: evidence for cognitive deficits in adult female offspring. Neurotoxicol. Teratol. 56 47–54. 10.1016/j.ntt.2016.06.002 27286749 PMC5292038

[B55] Ryait H. Bermudez-Contreras E. Harvey M. Faraji J. Mirza Agha B. Gomez-Palacio Schjetnan A. (2019). Data-driven analyses of motor impairments in animal models of neurological disorders. PLoS Biol. 17:e3000516. 10.1371/journal.pbio.3000516 31751328 PMC6871764

[B56] Sacrey L.-A. R. Bryson S. Zwaigenbaum L. Brian J. Smith I. M. Roberts W. (2018). The autism parent screen for infants: predicting risk of autism spectrum disorder based on parent-reported behavior observed at 6–24 months of age. Autism 22 322–334. 10.1177/1362361316675120 29671640

[B57] Sacrey L. A. R. Raza S. Armstrong V. Brian J. A. Kushki A. Smith I. M. (2020). Physiological measurement of emotion from infancy to preschool: a systematic review and meta-analysis. Brain Behav. 11:e01989. 33336555 10.1002/brb3.1989 PMC7882167

[B58] Samek W. Binder A. Montavon G. Lapuschkin S. Muller K. R. (2017). Evaluating the Visualization of What a Deep Neural Network Has Learned. IEEE Trans. Neur. Netw. Lear. 28 2660–2673. 10.1109/tnnls.2016.2599820 27576267

[B59] Samek W. Wiegand T. Muller K. R. (2018). Explanable Artificial inteligence: understanding, visualizing and interpreting deep learning models. ITU J. 1 39–48.

[B60] Schamhardt H. C. van den Bogert A. J. Hartman W. (1993). Measurement techniques in animal locomotion analysis. Acta Anat. 146 123–129. 10.1159/000147433 8470454

[B61] Schjetnan A. G. Luczak A. (2011). Recording Large-scale Neuronal Ensembles with Silicon Probes in the Anesthetized Rat. J. Vis. Exp. 2011:3282. 10.3791/3282 PMC3227202 22042361

[B62] Schjetnan A. G. P. Gidyk D. Metz G. A. S. Luczak A. (2019). Direct Current Stimulation Improves Limb Use After Stroke by Enhancing Inter-hemispheric Coherence. Acta Neurobiol. Exp. 79 290–301. 31587021

[B63] Shrikumar A. Greenside P. Kundaje A. (2017). “Learning Important Features Through Propagating Activation Differences” in Proceedings of the 34th International Conference on Machine Learning. eds Doina P. Yee Whye T. (United States: ACM Digital Library). 3145–3153.

[B64] Singer H. S. (2009). “Motor stereotypies” in Seminars in Pediatric Neurology. (Netherlands: Elsevier). 77–81. 10.1016/j.spen.2009.03.008 19501335

[B65] Srinivasan V. Lapuschkin S. Hellge C. Muller K. R. Samek W. (2017). Interpretable Human Action Recognition in Compressed Domain. United States: IEEE.

[B66] Sturman O. von Ziegler L. Schläppi C. Akyol F. Privitera M. Slominski D. (2020). Deep learning-based behavioral analysis reaches human accuracy and is capable of outperforming commercial solutions. Neuropsychopharmacology 45 1942–1952. 10.1038/s41386-020-0776-y 32711402 PMC7608249

[B67] Szegedy C. Liu W. Jia Y. Q. Sermanet P. Reed S. Anguelov D. (2015). “Going Deeper with Convolutions” in 2015 Ieee Conference on Computer Vision and Pattern Recognition (Cvpr). (United States: IEEE). 1–9.

[B68] Szegedy C. Vanhoucke V. Ioffe S. Shlens J. Wojna Z. (2016). Rethinking the Inception Architecture for Computer Vision. New York: Cornell University. 2818–2826.

[B69] Vassoler F. M. Byrnes E. M. Pierce R. C. (2014). The impact of exposure to addictive dugs on future generations: physiological and behavioral effects. Neuropharmacology 76 269–275. 10.1016/j.neuropharm.2013.06.016 23810828 PMC3864776

[B70] Wiltschko A. B. Johnson M. J. Iurilli G. Peterson R. E. Katon J. M. Pashkovski S. L. (2015). Mapping Sub-Second Structure in Mouse Behavior. Neuron 88 1121–1135. 10.1016/j.neuron.2015.11.031 26687221 PMC4708087

[B71] Wolgin D. L. (2012). Amphetamine stereotypy, the basal ganglia, and the “selection problem”. Behav. Brain Res. 231 297–308. 10.1016/j.bbr.2011.11.003 22101067

[B72] Wolraich M. L. Hagan J. F. Allan C. Chan E. Davison D. Earls M. (2019). Clinical practice guideline for the diagnosis, evaluation, and treatment of attention-deficit/hyperactivity disorder in children and adolescents. Pediatrics 144:e20192528. 10.1542/peds.2019-2528 31570648 PMC7067282

[B73] Yohn N. L. Bartolomei M. S. Blendy J. A. (2015). Multigenerational and transgenerational inheritance of drug exposure: the effects of alcohol, opiates, cocaine, marijuana, and nicotine. Prog. Biophys. Mol. Biol. 118 21–33. 10.1016/j.pbiomolbio.2015.03.002 25839742 PMC4459901

[B74] Zhou F. Duh H. B. L. Billinghurst M. (2008). Trends in Augmented Reality Tracking, Interaction and Display: a Review of Ten Years of ISMAR. United States: IEEE.

[B75] Zhu J. Lee K. P. Spencer T. J. Biederman J. Bhide P. G. (2014). Transgenerational transmission of hyperactivity in a mouse model of ADHD. J. Neurosci. 34 2768–2773. 10.1523/jneurosci.4402-13.2014 24553919 PMC3931498

