# Supplementary material for: A Neural Network Reveals Motoric Effects of Maternal Preconception Exposure to Nicotine on Rat Pup Behavior: A New Approach for Movement Disorders Diagnosis
Source: Front Neurosci. 2021 Jul 20;15:686767. doi: 10.3389/fnins.2021.686767 (PMC8329707; doi:10.3389/fnins.2021.686767)
Supplement: Supplementary Figure 1 — (A) Principal component (PC) space of behavioral measures. Each dot represents a single animal with MPNE group denoted in blue and control group in orange. Note that more control animals have high values of PC1 in comparison with the nicotine animals. To investigate this, we calculated the correlations between principal components and movement measures (B). We found that the first principal component has the largest correlation with the “Novel” followed by the “Total” measure. This indicates that control animals have a greater tendency to explore new places (i.e., enter a greater number of unique squares) as well as to explore more (i.e., enter more squares overall) than MPNE animals. [file Data_Sheet_1.docx]

# Supplementary Materials

## Suppl. Text 1. Applying machine learning algorithms on movement measures

We used five different machine learning methods (logistic regression; decision tree; random forest; k-nearest neighbors; support vector machine) to see how well the movement measures could discriminate between the two groups of animals. In the k-nearest method, we tried a different number of nearest neighbors to find the best accuracy. The best accuracy was found for k = 25 (‘k’ is the number of neighbors). In random forest algorithm, the best accuracy was obtained with the number of ensembles n = 50. In SVM, the accuracy with its default parameters is 58%. However, we used grid search for tuning the hyper parameters (C and Gamma) as well as the kernel type to find the best accuracy. The best accuracy was found for C =1, Gamma = 0.01, and polynomial kernel with degree 2.


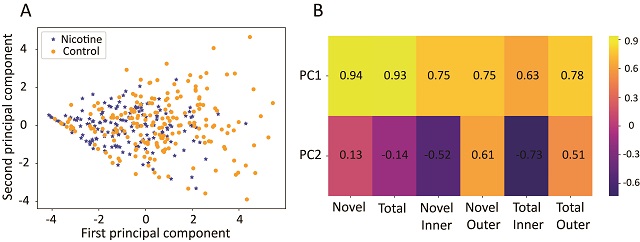


**Suppl. Fig. 1**: (A) Principal component (PC) space of behavioral measures. Each dot represents a single animal with MPNE group denoted in blue and control group in orange. Note that more control animals have high values of PC1 in comparison with the nicotine animals. To investigate this, we calculated the correlations between principal components and movement measures (B). We found that the first principal component has the largest correlation with the “Novel” followed by the “Total” measure. This indicates that control animals have a greater tendency to explore new places (i.e. enter a greater number of unique squares) as well as to explore more (i.e. enter more squares overall) than MPNE animals.

**
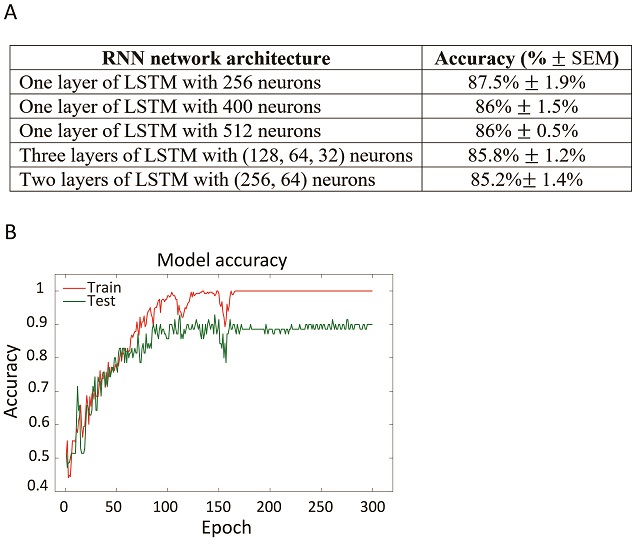
**

**Suppl. Fig. 2.** (A) Different architectures of recurrent neural network (RNN) tested. For this manuscript we selected the top network (one layer of LSTM with 256 neurons). However, all tested networks produced similar results, as shown in the table. The network performance was also robust to changes in video preprocessing. Specifically, down-sampling video by taking every 9th frame instead of every 10th frame (Methods) gave similar accuracy of ~89%. This shows that our network does not need fine tuning to outperform machine learning methods using expert selected movement measures.

(B) Sample learning curve from training top RNN with one layer of 256 long short-term memory (LSTM) (Hochreiter and Schmidhuber, 1997; Greff et al., 2017) units. Note that on training data we achieved 100% accuracy (red line), however on testing data accuracy was reduced (green line). This suggests that with larger dataset performance of the network may further improve.


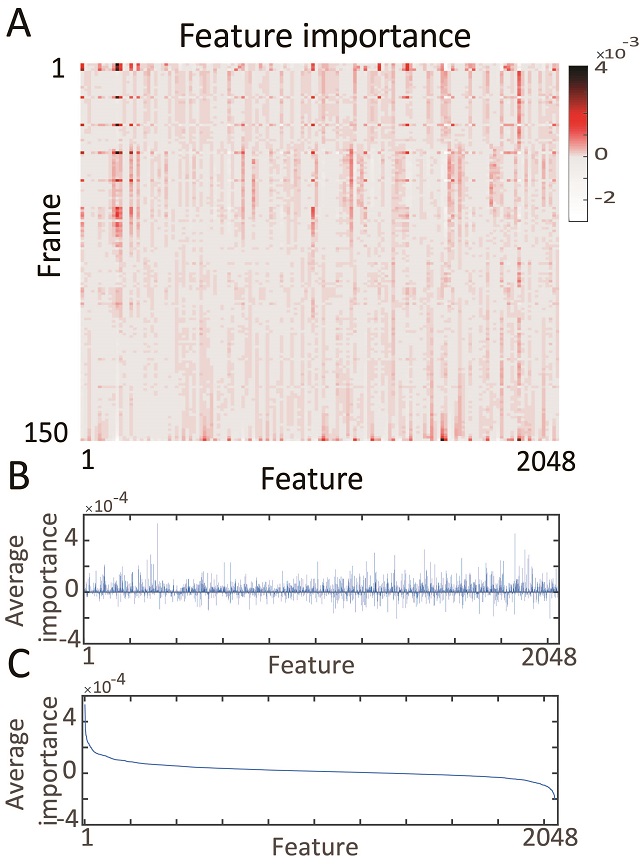


**Suppl. Fig. 3.** Most informative features for network decisions. (A) Average of feature’s importance over all videos, as shown in main text in Fig 3A. Considering reoccurring peaks in feature importance, it is apparent that the network is identifying periodic behavior, especially in the early 50 frames (~ 17 sec), to distinguish MPNE animals from control animals. Our RNN network was able to detect a repetitive pattern in the behavior because it is composed of the LSTM units which have memory and are specialized in identifying sequences of activity. (B) Average relevance (importance) of each 2048 features across all video data. Average relevance was obtained by averaging columns in the matrix shown in panel (A). (C) The same average feature importance as in (B), but sorted from highest to the lowest value. It illustrates that about 20 features had a disproportional effect on network decision making.


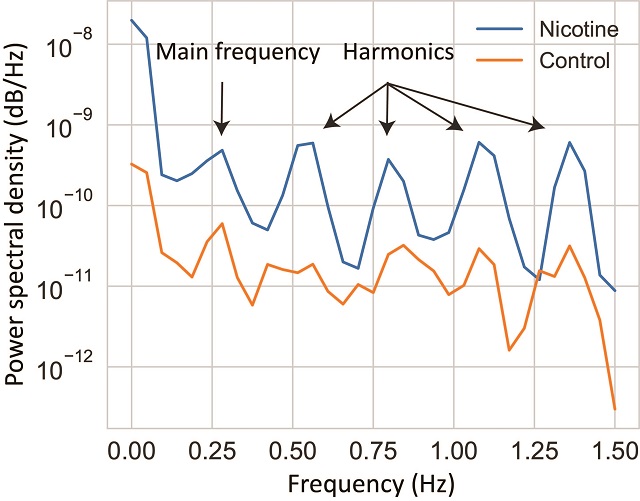


**Suppl. Fig. 4.** Power spectra analysis. In order to investigate periodic behavior shown in Fig. 3B, we calculated power spectra of the average feature importance. Blue and orange lines denote MPNE and control animals, respectively. Peak in power spectra at frequency of 0.27 Hz confirmed that video features oscillate with a period of 1/0.27=3.7s. Note that this periodic behavior was seen mostly in MPNE animals. This indicates that MPNE animals have much more stereotypical behavior, while control animals have more diverse and less repetitive movements.


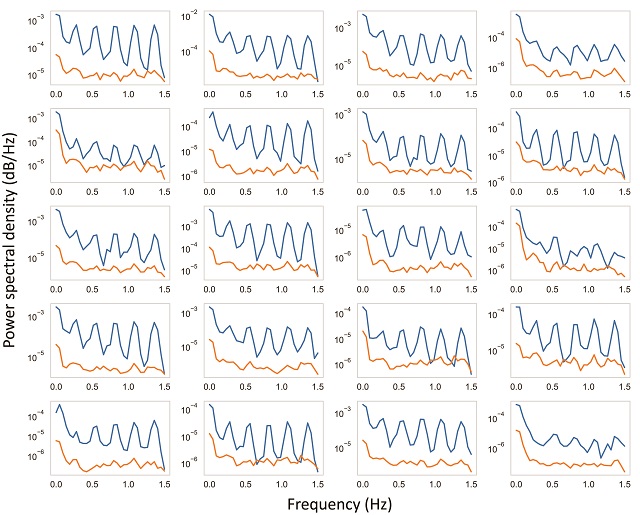


**Suppl. Fig. 5.** Power spectral analysis for 20 most important features. The blue and orange lines indicate MPNE and control animals, respectively. After identifying the most important features as illustrated in Suppl. Fig 4C, the power spectral for each of the 20 top features was calculated in each video. Then, we averaged spectra of each feature, separately for MPNE and control animals, which lead to the 20 graphs shown above. As can be seen, the periodic behavior with the frequency of about 0.27 Hz is clearly visible in nearly all of the features.


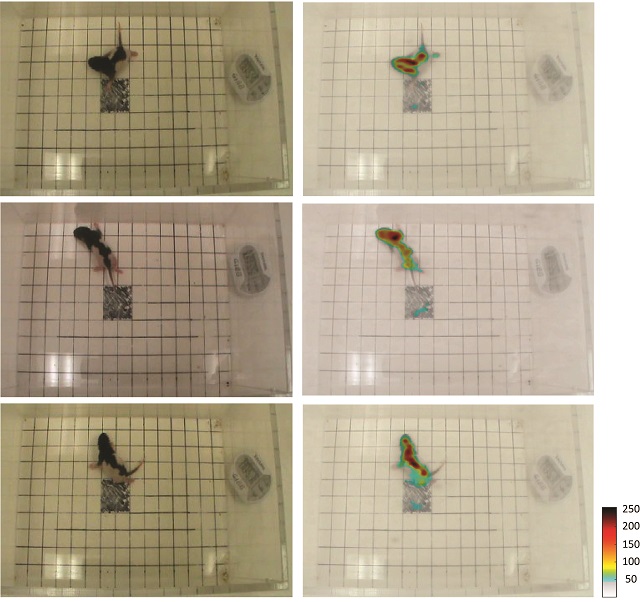


**Suppl. Fig. 6:** (Left column) Representative video frames, and the same frames with superimposed network focus (Right column). Red color scale denotes the most informative pixels used by the network to make decisions. This allowed us to verify that the network used features related to rat posture to discriminate control from MPNE animals. This analyses also ensures that the network does not ‘cheat’ by using spurious features like the clock display. To superimpose pixel importance on the frames, the values of importance were rescaled to range 50-250. The pixel importance was obtained by using LRP method described above.

Suppl. Text 2. Notes on knowledge extraction from neural networks.

Although deep neural networks have demonstrated impressive performance in complex machine learning tasks (Barnickel et al., 2009; Collobert et al., 2011; Le et al., 2011; Ciresan et al., 2012; Montavon et al., 2012; Ji et al., 2013; Szegedy et al., 2015; Krizhevsky et al., 2017) and are key components of many critical decision or predictive processes, they have the disadvantage of acting as a black box by not providing information about the internal reasoning underlying classification decisions (Bach et al., 2015; Lapuschkin et al., 2016; Samek et al., 2017; Srinivasan et al., 2017; Alber et al., 2019). In an attempt to overcome this shortcoming, multiple analysis methods have been proposed to explain predictions of complex non-linear classifiers in terms of input variables (Bach et al., 2015; Samek et al., 2017; Ancona et al., 2018; Alber et al., 2019). One of the most powerful methods in this respect is Layer-wise Relevance Propagation (LRP) (Bach et al., 2015), which we used here. LRP algorithms operate by propagating the prediction backward in the neural network and quantifying the “importance” of input components of classification decisions by attributing relevance scores to them. Video data contain both special (pixels) and temporal (frames) information, so the relevance data provide both special and temporal information about network decision making (Srinivasan et al., 2017; Samek et al., 2018). Special information showed us where the network was looking in the classification task (Srinivasan et al., 2017). This showed that the network is considering the difference in the behavior of control and MPNE animals in classification of video data rather than differences in irrelevant features that may vary between videos (e.g. position of the timer, orientation of the box, or glare from overhead lighting). Temporal information identifies the most relevant time points (Samek et al., 2018) in the behavior. Here, we present a general approach to analyzing behavioral video data by identifying the most important spatio-temporal features used by the network for animal classification. This allows us to uncover in a data-driven way the behavioral components most affected by MPNE.
